# Workload of Queueing Systems with Autocorrelated Service Times

**DOI:** 10.3390/e27030272

**Published:** 2025-03-05

**Authors:** Andrzej Chydzinski

**Affiliations:** Department of Computer Networks and Systems, Silesian University of Technology, Akademicka 16, 44-100 Gliwice, Poland; andrzej.chydzinski@polsl.pl

**Keywords:** queueing systems, performance evaluation, autocorrelated service times, workload distribution, average workload, workload entropy, time-dependent characteristic, steady-state characteristic

## Abstract

The queuing model with autocorrelated service times is studied with respect to workload, i.e., the time needed to serve all the customers in the queue. Specifically, new formulas for the probability density of workload, its tail, the average value, and entropy are derived and illustrated using numerical examples. Both time-dependent and steady-state cases are covered. It is also demonstrated that the average workload may reach surprisingly large values, exceeding several times the product of the average queue size and the average service time.

## 1. Introduction

In every queueing theory textbook, the study of the performance of a system begins with the queue size—its distribution and average value are usually derived first. The reason for this is perhaps that, in most real-life queueing systems, the queue size is the easiest aspect to observe and interpret.

Another important characteristic of queueing systems is workload, understood as the total time required to serve all the customers in the queue, i.e., the time needed to empty the system completely through the service process, assuming new arrivals are suspended.

In simple queueing systems, without autocorrelation in arrival or service processes, and with small variances of interarrival and service times, workload is roughly proportional to the queue size. Specifically, if the queue size is *X* and the average service time is $\bar{F}$, the workload is approximately $X\bar{F}$. In the M/M/1 queueing system, workload is exactly equal to $X\bar{F}$.

However, when the service times are autocorrelated, such intuitive calculations of workload can be completely inaccurate. As will be seen, workload can also be equal to $3X\bar{F}$ rather than $X\bar{F}$. In other words, knowledge of the queue size provides no information about the system workload when the service times are autocorrelated.

Because of that, in this paper, we will derive several formulas characterizing workload in the queueing model with autocorrelated service times. Specifically, formulas will be derived for the tail of the workload distribution, the probability density of workload, its entropy, and the average value. These formulas will encompass both time-dependent and steady-state workloads. The considered queueing model is universal in the meaning that the interarrival time distribution is general, whereas service times are modeled by the Markovian service process. This feature allows for an alignment of the autocorrelation function of the model with an empirical autocorrelation function. Finally, in addition to presenting new theorems and formulas, numerical examples will be provided to illustrate various workload-related characteristics and compare them with the average queue size.

There are several convincing reasons to study models with autocorrelated service times. For example, human-operated queueing systems may exhibit autocorrelation of service times caused by the operator’s tiredness, resulting in longer service times, or due to his/her learning process, which shortens the service times. Another reason for service autocorrelation in such systems could be the similarity of customers arriving at the queue. For instance, customers calling a call center about the same problem may require similar attendance times. In telecommunications, consecutive packet sizes can be correlated, leading to autocorrelated transmission times. In computing, autocorrelated task execution can result from resource sharing. Specifically, when a service is under heavy load caused by external tasks, it can decelerate the service in the queue of interest, leading to long, autocorrelated service times.

In short, the principal contributions of this study are as follows:

– a theorem on the tail of the distribution of workload at arbitrary time;
– a theorem on the probability density of workload at arbitrary time;
– a theorem on the average workload at arbitrary time;
– numeric examples of these characteristics, including a demonstration of counterintuitive values of workload when paired with the queue size.

The following organization applies to the rest of the article. In Section 2, the related work is reviewed, whereas in Section 3, the queueing model in question is formally defined. Section 4 is devoted to the derivation of the workload distribution. Its main results are Theorems 1 and 2, concerning the tail and the probability density of workload, respectively. In addition to that, the entropy of workload is obtained, basing on Theorems 1 and 2. In Section 5, the average workload is derived, with the final result of this section presented in Theorem 3. In Section 6, numeric examples are provided. These include sample calculations of the probability density, distribution tail, entropy, and average workload. Section 7 concludes the study with a few final remarks.

## 2. Related Work

To the author’s recognition, all the main results of this study, i.e., Theorems 1–3 and numerical examples, are new.

The vast majority of previous work on systems with Markovian service processes has focused solely on steady-state calculations.

Specifically, in [1], the queue size distribution in a model with a finite buffer is derived. In [2], the decay rates of large queue sizes in infinite-buffer models are investigated. In [3], the queue size and response time distributions are obtained for a model with a finite buffer and group service process. In [4], the performance of systems with finite and infinite buffers is evaluated using the queue size distribution along with the response time distribution. In [5], multiple servers with Markovian service processes are introduced. For such a model, the queue size distribution is obtained and accompanied by the idle and partly-idle period calculations, i.e., periods when every server is idle or when some servers are idle, respectively. In [6], the limit of the customer blocking probability, as the buffer size approaches infinity, is studied.

Study [7] is devoted to a differently defined service process. Namely, after an idle period, the service process returns to a fixed phase. Thus, in this model, the autocorrelation of service times isolated by an idle period is broken. Typically, in Markovian service processes, it is assumed that the correlation of service times is preserved through the idle period, i.e., the service phase remains the same as it was just before the idle period. However, both approaches may be useful in practice.

In [8], the queue with group arrivals and a finite buffer is investigated under two possible acceptance rules. The first assumes that, when the buffer is nearly full, part of the arriving group of customers can be accepted, filling the buffer completely, while the remainder is blocked. The second rule blocks the entire group in such cases. Several performance characteristics are derived for both variants, i.e., the queue size and waiting time distributions, and the blocking probability for the first, the last, and an arbitrary customer in an arriving group.

In [9], the model with group services and an infinite buffer is investigated with respect to the queue size distribution at arrival and arbitrary times. Additionally, the response time is calculated. In [10], roots of the characteristic equation are used to derive the queue size distribution for a model with single services and an infinite buffer. In [11], the response time and the autocorrelation of inter-departure times are studied in the infinite buffer model.

Two articles [12,13] focus on models with group arrivals and infinite buffers. One [12] examines the queue size along with idle and busy periods, while the other [13] investigates the distribution of waiting times.

The model with group arrivals is studied again in [14], but under a finite-buffer assumption. For this model, the average waiting time of a random customer, as well as the first and last customers in a group, is obtained. Additionally, the probability of blocking an arbitrary customer and the probability of blocking *k* or more consecutive customers during a busy period are derived. In [15], the model with group services and a finite buffer is revisited, aiming to optimize a profit function that combines all key performance characteristics, e.g., average queue size, waiting time, blocking probability, and others.

Article [16] compares two models with Markovian service processes regarding their queue sizes. In the first model, the service phase remains constant during idle periods, whereas in the second, it changes according to a Markov chain. In both models, group service is assumed.

The only published study involving the time-dependent analysis of a model with a Markovian service process [17] is devoted solely to the queue size. As will be seen, knowledge of the queue size provides no insight into workload when service times are autocorrelated.

Lastly, it is worth noting that the references discussed above focus on models with general interarrival distributions, as is the case here. Other studies with Markovian service processes are based either on specific interarrival distributions (e.g., Poisson, MAP, BMAP) or on discrete queueing systems with discrete-time Markovian service, e.g., [18,19,20,21,22,23,24,25,26,27].

## 3. Model

The model of interest is the model of a queueing system with a single server. Specifically, the next customer arrives to the queue after time is distributed according to G(t), where G(t) is some distribution function on (0,∞), not further specified, with the average value: $\bar{G}$,

(1) $$\bar{G}=\int_{0}^{\infty }\left[1-G\left(t\right)\right]dt<\infty ,$$

and the Laplace–Stieltjes transform,

(2) $$g\left(s\right)=\int_{0}^{\infty }e^{-sx}dG\left(x\right).$$

All interarrival intervals are mutually independent.

The queue of customers is formed following the arrival order and served from the head.

Service times of customers are mutually dependent and follow the Markovian service process [4]. Namely, there is an underlying random process S(t), called the service phase at time *t*. S(t) may assume *M* values, 1,…,M. To fully parameterize S(t), we need two matrices of size M×M, $L_{0}$, and $L_{1}$, such that $L_{0}$ is negative on the diagonal and nonnegative off the diagonal, and $L_{1}$ is nonnegative, while $L=L_{0}+L_{1}$ is a transition-rate matrix, with every row summing to 0.

The evolution of S(t) produces various service times of different customers, in the interdependent manner, as follows. When the number of customers in the system at some *t* is non-zero and S(t)=i, then, immediately after *t*, at t+Δ, the service phase may jump to *j* with probability ${\left(L_{0}\right)}_{ij}\Delta +o\left(\Delta \right)$, and the ongoing service of a customer is continued, or the phase may jump to *j* with probability ${\left(L_{1}\right)}_{ij}\Delta +o\left(\Delta \right)$, and the present service is completed, so the customer is departed from the system.

If the number of customers in the system becomes zero after a departure, then the evolution of the service phase is terminated for the whole idle period. Upon a new customer arrival after that period, S(t) starts evolving again according to the rule given in the previous paragraph, beginning with the same phase it had just before the idle period. (Note that from this definition it follows that S(t) is not a Markov process).

The system volume is finite and equal to *B* customers, including the one being served. If, at the time of a new arrival, there are *B* customers in the system, the new customer is blocked. Specifically, he/she leaves unserved the system and never arrives again.

It is easy to check that the average service time is

(3) $$\bar{F}=\frac{1}{\pi L_{1}e},$$

where **e** denotes the column vector of 1’s, whereas π denotes the stationary vector of transition-rate matrix L.

The correlation of two arbitrary service times can be computed in the same way as in the case of Markovian arrival processes (see, e.g., [28,29]). Specifically, we have

(4) $$Corr\left(n\right)=Corr\left(s_{k},s_{k+n}\right)=-\frac{\alpha L_{0}^{-1}K\left(K^{n-1}-e\alpha \right)L_{0}^{-1}Ke}{2\pi L_{1}e\pi L_{0}^{-1}e+{\left(\pi L_{1}e\right)}^{2}},$$

where $s_{k}$ is the *k*-th service time, $s_{k+n}$ is the service time which follows after other n−1 completed services, α is the stationary vector for transition-rate matrix K−I, I is the identity matrix, and

(5) $$K=-L_{0}^{-1}\left(L-L_{0}\right).$$

In the derivations of the next section, a vital role will be undertaken by two characteristics of the service process, $p_{j,k,l}\left(x\right)$ and $q_{j,k,l}\left(x\right)$. The former, $p_{j,k,l}\left(x\right)$, is defined as the probability that *l* customers are served in period (0,x), and the service phase is *k* at the end of the *l*-th service time, assuming X(0)>l and S(0)=j. The latter, $q_{j,k,l}\left(x\right)$, is defined as the probability that *l* customers are served in (0,x), and the service phase is *k* at time *x*, assuming X(0)>l and S(0)=j. It will subsequently be shown how these two characteristics can be calculated.

By X(t), we will represent the queue size at *t*, including the customer under service, if applicable. We also adopt the convention that every time the phrase “queue size” is used, it means “including the customer under service, if any”.

Lastly, it is presumed that the first arrival time has distribution G(t), i.e., the arrival process is not shifted at the time origin.

## 4. Workload Distribution and Entropy

Let ω(t) denote workload at time *t*, i.e., the time needed to empty the system completely by the service process, assuming there were no new arrivals after *t*. Obviously, ω(t) is a random variate, so it has to be characterized by the probability distribution. For technical reasons, it is easier to start with calculations of the tail of the distribution of ω(t), namely,

(6) $$W_{m,j}\left(t,u\right)=P\left\{\omega \left(t\right)>u|X\left(0\right)=m,S\left(0\right)=j\right\}.$$

Having the tail, we can procure the distribution function of the workload:

(7) $${\bar{W}}_{m,j}\left(t,u\right)=1-W_{m,j}\left(t,u\right).$$

We start with constructing integral equations for $W_{m,j}\left(t,u\right)$. Firstly, for an initially empty system, X(0)=0, and any j∈{1,…,M}, we have

(8) $$\begin{array}{c} W_{0,j}\left(t,u\right)=\int_{0}^{t}W_{1,j}\left(t-x,u\right)dG\left(x\right). \end{array}$$

This system is obtained by conditioning upon the time of arrival of the first customer, *x*. Indeed, when no customer is present, the service phase remains the same, i.e., *j*. When the first customers arrives, the service begins with this phase and a queue size of 1. Thus, the new tail of the workload distribution at time t−x is $W_{1,j}$.

Secondly, for an initially full system, X(0)=B, and any j∈{1,…,M}, we get

(9) $$\begin{array}{cc} W_{B,j}\left(t,u\right)= & \sum_{k=1}^{M}\int_{0}^{t}q_{j,k,0}\left(x\right)W_{B,k}\left(t-x,u\right)dG\left(x\right)\\ \; & +\sum_{l=1}^{B-1}\sum_{k=1}^{M}\int_{0}^{t}q_{j,k,l}\left(x\right)W_{B-l+1,k}\left(t-x,u\right)dG\left(x\right)\\ \; & +\sum_{k=1}^{M}\int_{0}^{t}p_{j,k,B}\left(x\right)W_{1,k}\left(t-x,u\right)dG\left(x\right)\\ \; & +\left(1-G\left(t\right)\right)\sum_{l=0}^{B-1}\sum_{k=1}^{M}q_{j,k,l}\left(t\right)\sum_{a=0}^{B-l-1}\sum_{i=1}^{M}q_{k,i,a}\left(u\right). \end{array}$$

The first integral of (9) handles the condition of zero services completed in (0,x). Under such a condition, the tail of the workload distribution at time t−x is $W_{B,k}$ because the customer arriving at *x* is blocked. The second integral of (9) handles the condition of *l* services completed in (0,x), where 0<l<B. Under such a condition, the size of the queue is reduced by time *x*, but not to zero. The new tail of the workload distribution at t−x is $W_{B-l+1,k}$ because the customer arriving at *x* is allowed to the queue. The third integral of (9) handles the condition of *B* services completed in (0,x), which makes the system empty by *x*. Thus, counting the new customer arriving at *x*, the new tail of the workload distribution at t−x is $W_{l,k}$. The last component of (9) handles the condition of no new arrivals by *t*. Specifically, to have a non-zero tail of the workload distribution at *t* under this condition, the system must not become empty by *t*, which happens with the following probability:

(10) $$\sum_{l=0}^{B-1}\sum_{k=1}^{M}q_{j,k,l}\left(t\right).$$

Furthermore, under such condition, we have

(11) $$P\left\{\omega \left(t\right)>u\right\}=\sum_{a=0}^{B-l-1}\sum_{i=1}^{M}q_{k,i,a}\left(u\right),$$

which completes the explanation of (9).

For a partially occupied system, 0<X(0)=m<B, and any j∈{1,…,M}, we obtain

(12) $$\begin{array}{cc} W_{m,j}\left(t,u\right)=\; & \sum_{l=0}^{m-1}\sum_{k=1}^{M}\int_{0}^{t}q_{j,k,l}\left(x\right)W_{m-l+1,k}\left(t-x,u\right)dG\left(x\right)\\ \; & +\sum_{k=1}^{M}\int_{0}^{t}p_{j,k,m}\left(x\right)W_{1,k}\left(t-x,u\right)dG\left(x\right)\\ \; & +\left(1-G\left(t\right)\right)\sum_{l=0}^{m-1}\sum_{k=1}^{M}q_{j,k,l}\left(t\right)\sum_{a=0}^{m-l-1}\sum_{i=1}^{M}q_{k,i,a}\left(u\right). \end{array}$$

Equation (12) can be explained in the same way as (9), with the exception that (12) has no analog of the first part of (9). This is because, in the case of X(0)<B, no customer can be blocked by the first arrival time, *x*.

Denoting

(13) $$w_{m,j}\left(s,u\right)=\int_{0}^{\infty }e^{-sx}W_{m,j}\left(x,u\right)dx,$$

(14) $$x_{j,k,l}\left(s\right)=\int_{0}^{\infty }e^{-sx}p_{j,k,l}\left(x\right)dG\left(x\right),$$

(15) $$y_{j,k,l}\left(s\right)=\int_{0}^{\infty }e^{-sx}q_{j,k,l}\left(x\right)dG\left(x\right),$$

(16) $$c_{j,k,l}\left(s\right)=\int_{0}^{\infty }e^{-sx}q_{j,k,l}\left(x\right)\left[1-G(x)\right]dx,$$

we can restate Equation (8) as

(17) $$\begin{array}{c} w_{0,j}\left(s,u\right)=g\left(s\right)w_{1,j}\left(s,u\right), \end{array}$$

while (9) can be restated as

(18) $$\begin{array}{cc} w_{B,j}\left(s,u\right)= & \sum_{k=1}^{M}y_{j,k,0}\left(s\right)w_{B,k}\left(s,u\right)+\sum_{l=1}^{B-1}\sum_{k=1}^{M}y_{j,k,l}\left(s\right)w_{B-l+1,k}\left(s,u\right)\\ \; & +\sum_{k=1}^{M}x_{j,k,B}\left(s\right)w_{1,k}\left(s,u\right)+\sum_{l=0}^{B-1}\sum_{a=0}^{B-l-1}\sum_{k=1}^{M}\sum_{i=1}^{M}c_{j,k,l}\left(s\right)q_{k,i,a}\left(u\right), \end{array}$$

and (12) as

(19) $$\begin{array}{cc} w_{m,j}\left(s,u\right)= & \sum_{l=0}^{m-1}\sum_{k=1}^{M}y_{j,k,l}\left(s\right)w_{m-l+1,k}\left(s,u\right)+\sum_{k=1}^{M}x_{j,k,m}\left(s\right)w_{1,k}\left(s,u\right)\\ \; & +\sum_{l=0}^{m-1}\sum_{a=0}^{m-l-1}\sum_{i=1}^{M}\sum_{k=1}^{M}c_{j,k,l}\left(s\right)q_{k,i,a}\left(u\right),\;\;\;\;0<m<B. \end{array}$$

Employing the vector

(20) $$w_{m}\left(s,u\right)={\left[w_{m,1}\left(s,u\right),\ldots ,w_{m,M}\left(s,u\right)\right]}^{T},$$

and matrices

(21) $$X_{l}\left(s\right)={\left[x_{j,k,l}\left(s\right)\right]}_{j=1,\ldots ,M;k=1,\ldots ,M},$$

(22) $$Y_{l}\left(s\right)={\left[y_{j,k,l}\left(s\right)\right]}_{j=1,\ldots ,M;k=1,\ldots ,M},$$

to (17), (18), and (19) yields

(23) $$\begin{array}{c} w_{0}\left(s,u\right)=g\left(s\right)w_{1}\left(s,u\right), \end{array}$$

(24) $$\begin{array}{cc} w_{B}\left(s,u\right)= & Y_{0}\left(s\right)w_{B}\left(s,u\right)+\sum_{l=1}^{B-1}Y_{l}\left(s\right)w_{B-l+1}\left(s,u\right)+X_{B}\left(s\right)w_{1}\left(s,u\right)+b_{B}\left(s,u\right) \end{array}$$

(25) $$\begin{array}{cc} w_{m}\left(s,u\right)= & \sum_{l=0}^{m-1}Y_{l}\left(s\right)w_{m-l+1}\left(s,u\right)+X_{m}\left(s\right)w_{1}\left(s,u\right)+b_{m}\left(s,u\right),\;\;\;\;0<m<B, \end{array}$$

respectively, with

(26) $$b_{m}\left(s,u\right)=\sum_{l=0}^{m-1}C_{l}\left(s\right)\sum_{a=0}^{m-l-1}Q_{a}\left(u\right)e,$$

(27) $$Q_{l}\left(u\right)={\left[q_{j,k,l}\left(u\right)\right]}_{j=1,\ldots ,M;k=1,\ldots ,M},$$

(28) $$C_{l}\left(s\right)={\left[c_{j,k,l}\left(s\right)\right]}_{j=1,\ldots ,M;k=1,\ldots ,M}.$$

Before presenting the solution of system (23)–(25), it ought to be underlined that all the coefficient matrices in (23)–(25) can be calculated numerically. Specifically, $Y_{l}\left(s\right)$, $C_{l}\left(s\right)$, and $Q_{l}\left(u\right)$ can be computed using the uniformization algorithm [30,31], whereas $X_{l}\left(s\right)$ can be obtained using the formulas proven in [17], namely,

(29) $$\begin{array}{c} X_{l}\left(s\right)=C_{l-1}\left(s\right)L_{1}-sH_{l}\left(s\right),\;\;\;\;\;\;l\geq 1, \end{array}$$

with

(30) $$\begin{array}{c} H_{1}\left(s\right)=C_{0}\left(s\right)L_{0}^{-1}L_{1}-\bar{g}\left(s\right)L_{0}^{-1}L_{1},\;\;\;\bar{g}\left(s\right)=\int_{0}^{\infty }e^{-sx}\left(1-G\left(x\right)\right)dx, \end{array}$$

(31) $$\begin{array}{c} H_{l+1}\left(s\right)=C_{l}\left(s\right)L_{0}^{-1}L_{1}-H_{l}\left(s\right)L_{0}^{-1}L_{1},\;\;\;\;\;l\geq 1. \end{array}$$

We can now move to finding the solutions of (23)–(25). Specifically, applying the lemma of [32], p. 200, we realize that the solution of (25) is

(32) $$w_{m}\left(s,u\right)=R_{m}\left(s\right)c\left(s,u\right)+\sum_{l=1}^{m}R_{m-l}\left(s\right)a_{l}\left(s,u\right),$$

where vector c(s,u) is unknown, but independent of *m*, whereas

(33) $$a_{m}\left(s,u\right)=Y_{m}\left(s\right)w_{1}\left(s,u\right)-X_{m}\left(s\right)w_{1}\left(s,u\right)-b_{m}\left(s,u\right),$$

(34) $$R_{0}\left(s\right)=0,\;\;\;\;R_{1}\left(s\right)=Y_{0}^{-1}\left(s\right),$$

(35) $$\begin{array}{c} R_{m+1}\left(s\right)=Y_{0}^{-1}\left(s\right)(R_{m}\left(s\right)-\sum_{l=0}^{m}Y_{l+1}\left(s\right)R_{m-l}\left(s\right)),\;\;\;\;m>0, \end{array}$$

and 0 represents the square zero matrix. Inserting m=1 into (32) yields

(36) $$c\left(s,u\right)=Y_{0}\left(s\right)w_{1}\left(s,u\right).$$

Then, using (32) together with (33) and (36), we have

(37) $$w_{m}\left(s,u\right)=E_{m}\left(s\right)w_{1}\left(s,u\right)-\sum_{l=1}^{m}R_{m-l}\left(s\right)b_{l}\left(s,u\right),\;\;\;\;m>0,$$

with

(38) $$E_{m}\left(s\right)=R_{m}\left(s\right)Y_{0}\left(s\right)+\sum_{l=1}^{m}R_{m-l}\left(s\right)Y_{l}\left(s\right)-\sum_{l=1}^{m}R_{m-l}\left(s\right)X_{l}\left(s\right).$$

What is left to find is the unknown $w_{1}\left(s,u\right)$ occurring in (37). After that, (37) will be fully solved. Fortunately, $w_{1}\left(s,u\right)$ can be found by utilizing (37) with m=B and leveraging (24) in addition to that. The final results can be gathered as follows.

**Theorem** **1.** *The transform of the tail of the workload distribution in the model with a Markovian service process is*

(39) $$\begin{array}{cc} \; & w_{0}\left(s,u\right)=\\ \; & g\left(s\right)A\left(s\right)\left(\sum_{l=1}^{B-1}Y_{l}\left(s\right)\;\sum_{k=1}^{B-l+1}\;R_{B-l+1-k}\left(s\right)b_{k}\left(s,u\right)+\left(Y_{0}\left(s\right)-I\right)\;\sum_{k=1}^{B}R_{B-k}\left(s\right)b_{k}\left(s,u\right)-b_{B}\left(s,u\right)\right), \end{array}$$

(40) $$w_{1}\left(s,u\right)=w_{0}\left(s,u\right)/g\left(s\right),$$

(41) $$w_{m}\left(s,u\right)=E_{m}\left(s\right)w_{0}\left(s,u\right)/g\left(s\right)-\sum_{k=1}^{m}R_{m-k}\left(s\right)b_{k}\left(s,u\right),\;\;\;\;1<m\leq B,$$

*where*

(42) $$A\left(s\right)={\left(\sum_{l=1}^{B-1}Y_{l}\left(s\right)E_{B-l+1}\left(s\right)-E_{B}\left(s\right)+Y_{0}\left(s\right)E_{B}\left(s\right)+X_{B}\left(s\right)\right)}^{-1},$$

*while $Y_{l}\left(s\right)$, $R_{l}\left(s\right)$, $b_{k}\left(s,u\right)$, $E_{m}\left(s\right)$, and $X_{B}\left(s\right)$ are given in (15), (35), (26), (38), and (29), respectively.*

Now we can proceed to calculations of the density of workload $f_{m,j}\left(t,u\right)$. By definition, we have

(43) $$f_{m,j}\left(t,u\right)=\frac{d{\bar{W}}_{m,j}\left(t,u\right)}{du},\;\;\;u>0.$$

(As we can see, this definition is restricted to u>0. The distribution of workload for u=0 will be discussed below).

Denote

(44) $$f_{m,j}^{*}\left(s,u\right)=\int_{0}^{\infty }e^{-sx}f_{m,j}\left(x,u\right)dx,$$

and

(45) $$f_{m}\left(s,u\right)={\left[f_{m,1}^{*}\left(s,u\right),\ldots ,f_{m,M}^{*}\left(s,u\right)\right]}^{T}.$$

We have obviously

(46) $$f_{m}\left(s,u\right)=-\frac{dw_{m}\left(s,u\right)}{du}.$$

Therefore, we have to compute the derivative of (39)–(41) with respect to *u*. In fact, variable *u* occurs in (39)–(41) only in $w_{k}\left(s,u\right)$ and $b_{k}\left(s,u\right)$, defined in (26). In order to differentiate (26), we can use the well-known formulas

(47) $$\frac{dQ_{l}\left(u\right)}{du}=Q_{l}\left(u\right)L_{0}+Q_{l-1}\left(u\right)L_{1},\;\;\;l\geq 1,$$

(48) $$Q_{0}\left(u\right)=e^{L_{0}u},\;\;\;\frac{dQ_{0}\left(u\right)}{du}=L_{0}e^{L_{0}u},$$

which give

(49) $$b_{m}^{\prime }\left(s,u\right)=\frac{db_{m}\left(s,u\right)}{du}=\sum_{l=0}^{m-1}C_{l}\left(s\right)\left(\sum_{a=1}^{m-l-1}Q_{a}\left(u\right)L_{0}+\sum_{a=1}^{m-l-1}Q_{a-1}\left(u\right)L_{1}+L_{0}e^{L_{0}u}\right)e.$$

The latter formula, accompanied with (39)–(41), delivers the final result.

**Theorem** **2.** *The transform of the probability density of the workload in the model with a Markovian service process is*

(50) $$\begin{array}{cc} \; & f_{0}\left(s,u\right)=\\ \; & -g\left(s\right)A\left(s\right)\left(\sum_{l=1}^{B-1}Y_{l}\left(s\right)\;\sum_{k=1}^{B-l+1}\;R_{B-l+1-k}\left(s\right)b_{k}^{\prime }\left(s,u\right)+\left(Y_{0}\left(s\right)\;-\;I\right)\;\sum_{k=1}^{B}\;R_{B-k}\left(s\right)b_{k}^{\prime }\left(s,u\right)\;-\;b_{B}^{\prime }\left(s,u\right)\right), \end{array}$$

(51) $$f_{1}\left(s,u\right)=f_{0}\left(s,u\right)/g\left(s\right),$$

(52) $$f_{m}\left(s,u\right)=E_{m}\left(s\right)f_{0}\left(s,u\right)/g\left(s\right)+\sum_{k=1}^{m}R_{m-k}\left(s\right)b_{k}^{\prime }\left(s,u\right),\;\;\;\;1<m\leq B,$$

*where $b_{m}^{\prime }\left(s,u\right)$ is given in (49).*

Now we can proceed to the derivation of the entropy of the workload. To accomplish that, we have to observe first that density $f_{m,j}\left(t,u\right)$ is defective; i.e., in general, it does not integrate to 1 with respect to *u*. This comes from the fact that if the queue is empty, which has a non-zero probability, then the workload is zero. Thus, the distribution of workload has an atom at u=0. The size of this atom, $P_{m,j}\left(t\right)$, is the following:

(53) $$P_{m,j}\left(t\right)=P\left\{\omega \left(t\right)=0|X\left(0\right)=m,S\left(0\right)=j\right\}=1-W_{m,j}\left(t,0\right).$$

In this way, the distribution of workload is in fact a mixture of a discrete distribution, which assumes only a value of 0, and a continues distribution, having probability density $f_{m,j}\left(t,u\right)/\left(1-P_{m,j}\left(t\right)\right)$. The two-point mixing distribution is the following: $\left(P_{m,j}\left(t\right),1-P_{m,j}\left(t\right)\right)$.

Denote by $h_{m,j}\left(t\right)$ the entropy of workload at time *t* given X(0)=m,S(0)=j, while $h_{m,j}^{d}\left(t\right)$ denotes the entropy of its discrete part, $h_{m,j}^{c}\left(t\right)$ denotes the entropy of its continues part, and $h_{m,j}^{*}\left(t\right)$ denotes the entropy of the mixing distribution. Adopting this notation yields

(54) $$h_{m,j}\left(t\right)=h_{m,j}^{*}\left(t\right)+P_{m,j}\left(t\right)h_{m,j}^{d}\left(t\right)+\left(1-P_{m,j}\left(t\right)\right)h_{m,j}^{c}\left(t\right),$$

(see, e.g., Formula (7) in [33]). Furthermore, we have

(55) $$h_{m,j}^{*}\left(t\right)=-P_{m,j}\left(t\right)\log\left(P_{m,j}\left(t\right)\right)-\left(1-P_{m,j}\left(t\right)\right)\log\left(1-P_{m,j}\left(t\right)\right),$$

(56) $$h_{m,j}^{d}\left(t\right)=0,$$

and

(57) $$\begin{array}{cc} h_{m,j}^{c}\left(t\right) & =-\int_{0}^{\infty }\frac{f_{m,j}\left(t,u\right)}{1-P_{m,j}\left(t\right)}\log\left(\frac{f_{m,j}\left(t,u\right)}{1-P_{m,j}\left(t\right)}\right)du\\ \; & =\log\left(1-P_{m,j}\left(t\right)\right)-\frac{1}{1-P_{m,j}\left(t\right)}\int_{0}^{\infty }f_{m,j}\left(t,u\right)\log\left(f_{m,j}\left(t,u\right)\right)du. \end{array}$$

Lastly, combining (54) with (55)–(57) and (53), we arrive at the final formula for the entropy of the workload:

(58) $$h_{m,j}\left(t\right)=-\left(1-W_{m,j}\left(t,0\right)\right)\log\left(1-W_{m,j}\left(t,0\right)\right)-\int_{0}^{\infty }f_{m,j}\left(t,u\right)\log\left(f_{m,j}\left(t,u\right)\right)du,$$

where $W_{m,j}\left(t,0\right)$ can be obtained from Theorem 1, while $f_{m,j}\left(t,u\right)$ can be obtained from Theorem 2.

Indeed, Theorems 1 and 2 can be used to calculate numerically $W_{m,j}\left(t,u\right)$ and $f_{m,j}\left(t,u\right)$ for a specific *t*, as well as in the steady state, for t=∞. To calculate the steady-state values, $W_{m,j}\left(\infty ,u\right)$ and $f_{m,j}\left(\infty ,u\right)$, we have to calculate numerically the limits s→0+ of $sw_{1}\left(s,u\right)$ and $sf_{1}\left(s,u\right)$, respectively, directly from Theorems 1 and 2. This follows from the well-known FVT theorem. To compute these characteristics at a specific *t*, we have to employ a numeric inversion formula for the transform [34].

## 5. Average Value

Define

(59) $$V_{m,j}\left(t\right)=E\left\{\omega \left(t\right)|X\left(0\right)=m,S\left(0\right)=j\right\}.$$

For an initially empty system, X(0)=0, and any j∈{1,…,M}, we have

(60) $$\begin{array}{c} V_{0,j}\left(t\right)=\int_{0}^{t}V_{1,j}\left(t-x\right)dG\left(x\right), \end{array}$$

which can be rationalized in the same manner as (8). For an initially full system, X(0)=B, and any j∈{1,…,M}, we obtain

(61) $$\begin{array}{cc} V_{B,j}\left(t\right)= & \sum_{k=1}^{M}\int_{0}^{t}q_{j,k,0}\left(x\right)V_{B,k}\left(t-x\right)dG\left(x\right)\\ \; & +\sum_{l=1}^{B-1}\sum_{k=1}^{M}\int_{0}^{t}q_{j,k,l}\left(x\right)V_{B-l+1,k}\left(t-x\right)dG\left(x\right)\\ \; & +\sum_{k=1}^{M}\int_{0}^{t}p_{j,k,B}\left(x\right)V_{1,k}\left(t-x\right)dG\left(x\right)\\ \; & +\left(1-G\left(t\right)\right)\sum_{l=0}^{B-1}\sum_{k=1}^{M}q_{j,k,l}\left(t\right)\sum_{a=0}^{B-l-1}\sum_{i=1}^{M}\int_{0}^{\infty }q_{k,i,a}\left(u\right)du. \end{array}$$

This equation differs from (9) only in the last term, which handles the condition of no new arrivals by *t*, which happens with probability $\sum_{l=0}^{B-1}\sum_{k=1}^{M}q_{j,k,l}\left(t\right)$. Under such a condition, the average workload at *t* equals $\sum_{a=0}^{B-l-1}\sum_{i=1}^{M}\int_{0}^{\infty }q_{k,i,a}\left(u\right)du$. Analogously, for a partially occupied system, we obtain

(62) $$\begin{array}{cc} V_{m,j}\left(t\right)=\; & \sum_{l=0}^{m-1}\sum_{k=1}^{M}\int_{0}^{t}q_{j,k,l}\left(x\right)V_{m-l+1,k}\left(t-x\right)dG\left(x\right)\\ \; & +\sum_{k=1}^{M}\int_{0}^{t}p_{j,k,m}\left(x\right)V_{1,k}\left(t-x\right)dG\left(x\right)\\ \; & +\left(1-G\left(t\right)\right)\sum_{l=0}^{m-1}\sum_{k=1}^{M}q_{j,k,l}\left(t\right)\sum_{a=0}^{m-l-1}\sum_{i=1}^{M}\int_{0}^{\infty }q_{k,i,a}\left(u\right)du,\;\;\;\;0<m<B. \end{array}$$

System (60)–(62) has the same layout as (8), (9), and (12), except for free terms. Due to that, it can be solved correspondingly, with obvious modifications. Using the notation

(63) $$v_{m,j}\left(s\right)=\int_{0}^{\infty }e^{-sx}V_{m,j}\left(x\right)dx,$$

(64) $$v_{m}\left(s\right)={\left[v_{m,1}\left(s\right),\ldots ,v_{m,M}\left(s\right)\right]}^{T},$$

the final solution has the following form.

**Theorem** **3.** *The transform of the average workload in the model with a Markovian service process is*

(65) $$\begin{array}{cc} v_{0}\left(s\right)= & g(s)A(s)\\ \; & \cdot \left(\sum_{l=1}^{B-1}Y_{l}\left(s\right)\sum_{k=1}^{B-l+1}R_{B-l+1-k}\left(s\right)d_{k}\left(s\right)+\left(Y_{0}\left(s\right)-I\right)\sum_{k=1}^{B}R_{B-k}\left(s\right)d_{k}\left(s\right)-d_{B}\left(s\right)\right), \end{array}$$

(66) $$v_{1}\left(s\right)=v_{0}\left(s\right)/g\left(s\right),$$

(67) $$v_{m}\left(s\right)=E_{m}\left(s\right)v_{0}\left(s\right)/g\left(s\right)-\sum_{k=1}^{m}R_{m-k}\left(s\right)d_{k}\left(s\right),\;\;\;\;1<m\leq B,$$

*where*

(68) $$d_{m}\left(s\right)=\sum_{l=0}^{m-1}C_{l}\left(s\right)\sum_{a=0}^{m-l-1}P_{a}e.$$

(69) $$P_{a}={\left[\int_{0}^{\infty }q_{j,k,a}\left(u\right)du\right]}_{j=1,\ldots ,M;k=1,\ldots ,M}.$$

## 6. Examples

In the numeric examples, the following parameter matrices of the Markovian service process are utilized:

$$L_{0}=\left[\begin{array}{ccc} -0.362348 & 0.011973 & 0.009551\\ 0.006509 & -2.739780 & 0.016561\\ 0.013568 & 0.010249 & -0.063200 \end{array}\right],$$

(70) $$L_{1}=\left[\begin{array}{ccc} 0.339476 & 0.000113 & 0.001235\\ 0.000405 & 2.716119 & 0.000186\\ 0.001879 & 0.000124 & 0.037380 \end{array}\right].$$

For these matrices, the average service time is $\bar{F}=1,$ while the 1-lag autocorrelation is Corr(1)=0.31.

The interearrival time has a hyperexponential distribution, with parameters (2, 0.4) and (0.75, 0.25), which gives the average interarrival time $\bar{G}=1$. Therefore, the system is fully loaded, with $\rho =\bar{F}/\bar{G}=1$. Finally, the system capacity is 20 customers.

**Figure 1:**
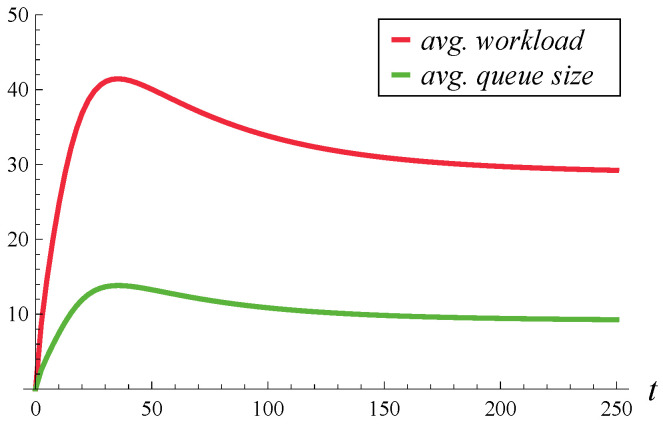
Average workload and queue size in time. X(0)=0, S(0)=1.

**Figure 2:**
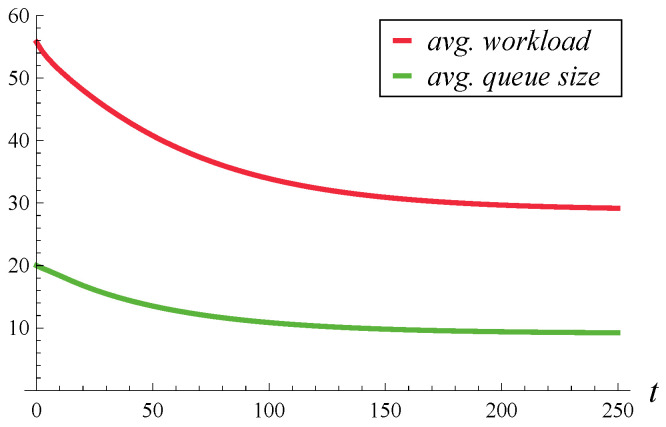
Average workload and queue size in time. X(0)=20, S(0)=1.

In Figure 1 and Figure 2, the evolution of the average workload and queue size over time is depicted. The average workloads were obtained from Theorem 3 proven here, while the average queue sizes were obtained from Theorem 2 of [17]. Figure 1 differs from Figure 2 by the initial system occupancy, which is 0 in Figure 1 and 20 in Figure 2. In both figures, the steady-state average queue size (i.e., the limit on the right-hand side) is 9.121, while the steady-state average workload is 28.834.

These numbers, and Figure 1 and Figure 2 in general, are quite surprising. Given that the average service time is 1, one might expect that the average queue sizes and average workloads should be close to each other, rather than differ by more than three times. Such a difference emphasizes the need for calculating the workload separately, as the queue sizes computed using the formulas of [17] provide no knowledge of workload.

The following explanation for such a large discrepancy between the average queue size and average workload can be proposed: In the Markovian service process, some service phases slow down the service process below the average, while others speed it up above the average. Moreover, when the autocorrelation is positive, as here, once the service is in a slow phase, it has a tendency to stay in this slow phase for a period of time, increasing the chances of a few long service times in a row. In addition to that, an arbitrarily chosen time *t* is more likely to be in a long service interval than in a short service interval. To put it differently, it is more likely to be in a slow service phase than in a fast service phase.

Therefore, an arbitrarily chosen *t* is not only more likely to be in a slow service phase, i.e., within a longer-than-average current service time, but also it is likely that a few subsequent services will also be long. This makes the tail of the workload quite heavy, much heavier than the tail of the queue size, which explains the discrepancy between the queue size and workload in Figure 1 and Figure 2.

**Figure 3:**
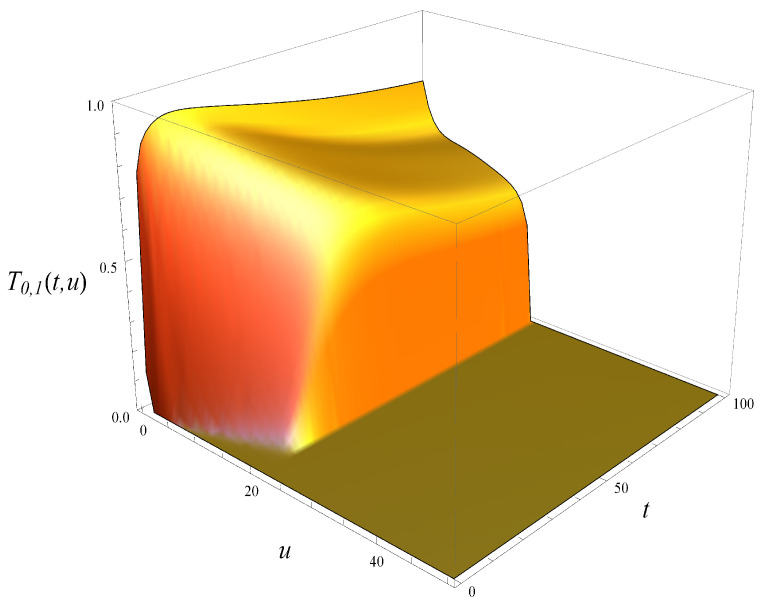
Tail of queue size distribution in time. X(0)=0, S(0)=1.

**Figure 4:**
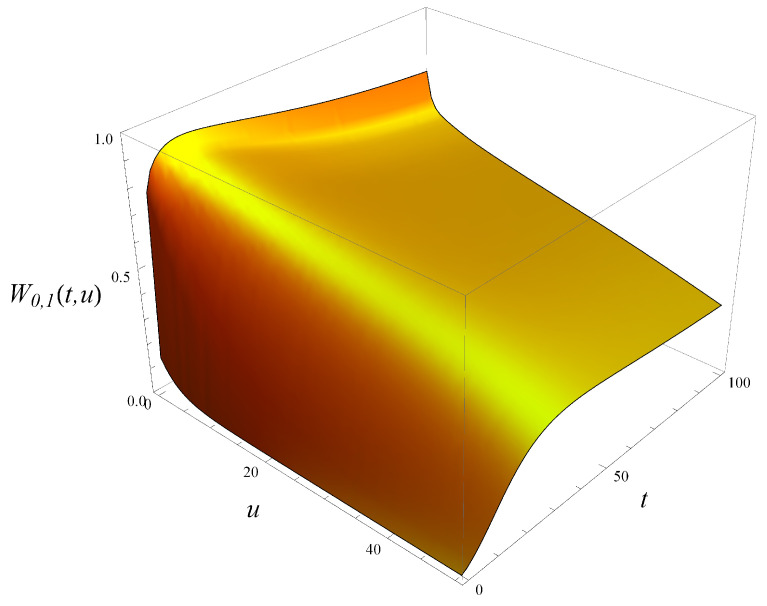
Tail of workload distribution in time. X(0)=0, S(0)=1.

To illustrate this further, in Figure 3 and Figure 4, both tails are depicted, i.e., the tail of queue size distribution defined as

(71) $$T_{m,j}\left(t,u\right)=P\left\{X\left(t\right)>u|X\left(0\right)=m,S\left(0\right)=j\right\},$$

and the tail of workload distribution defined in (6). As seen, the tail of workload in Figure 4 decreases much more slowly with *u* than the tail of the queue size, and this is true for every *t*.

Finally, the difference between the queue size and workload can also be observed from the information theory perspective. Namely, the entropy of the queue size distribution in the steady state equals to 2.11 nats. (This was calculated from Theorem 1 of [17]). On the other hand, the entropy of the workload distribution in the steady state is 3.63 nats, which was computed by means of Formula (58), with the help of Theorems 1 and 2. As seen, the entropy of the workload is much higher than the entropy of the queue size.

**Figure 5:**
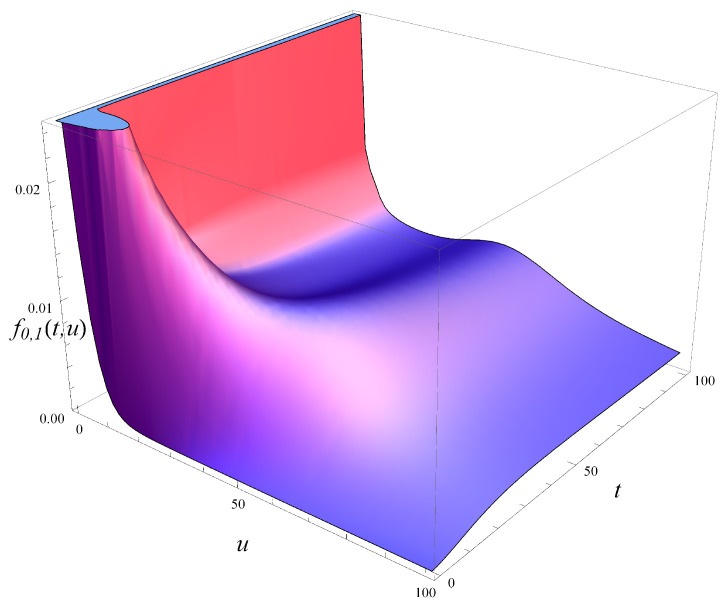
Probability density of workload in time. X(0)=0, S(0)=1.

**Figure 6:**
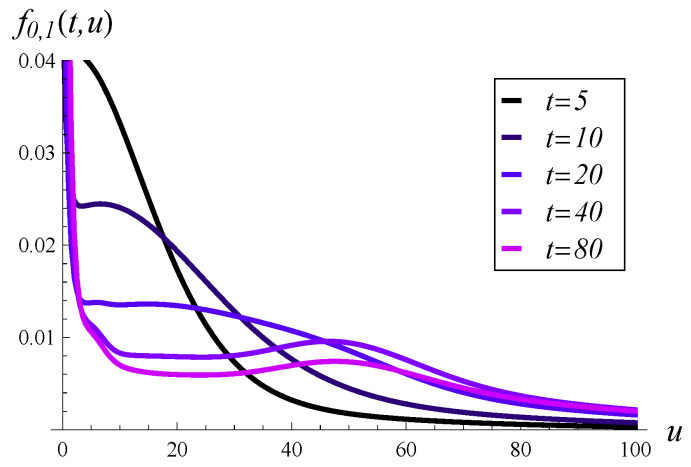
Probability density of workload in selected moments. X(0)=0, S(0)=1.

In Figure 5, the probability density of workload over time is depicted for an initially empty system. Five vertical slices of Figure 5, taken at different moments in time, are depicted in Figure 6. As seen in these figures, the probability mass is initially concentrated near u=0, but it quickly spreads out as time passes. For a low *t* of just 20, there is already a significant portion of the probability mass beyond u=100.

Note that the steady state has not yet been reached in Figure 5. As indicated in Figure 1, the steady state is reached at around t=250.

**Figure 7:**
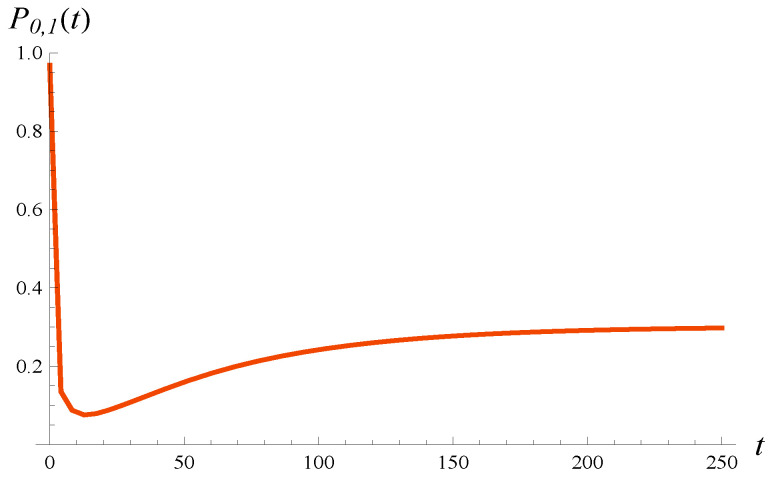
Probability that workload is zero versus time. X(0)=0, S(0)=1.

As discussed in Section 5, the densities of workload presented in Figure 5 and Figure 6 are defective; i.e., they do not integrate to 1. The missing probability is $P_{0,1}\left(t\right)$, which is the probability of zero workload at time *t*. This probability is depicted in Figure 7. Its steady-state value is 0.312. It is interesting that the evolution of $P_{0,1}\left(t\right)$ is not monotonic—it clearly has a minimum shortly after the system is activated.

**Figure 8:**
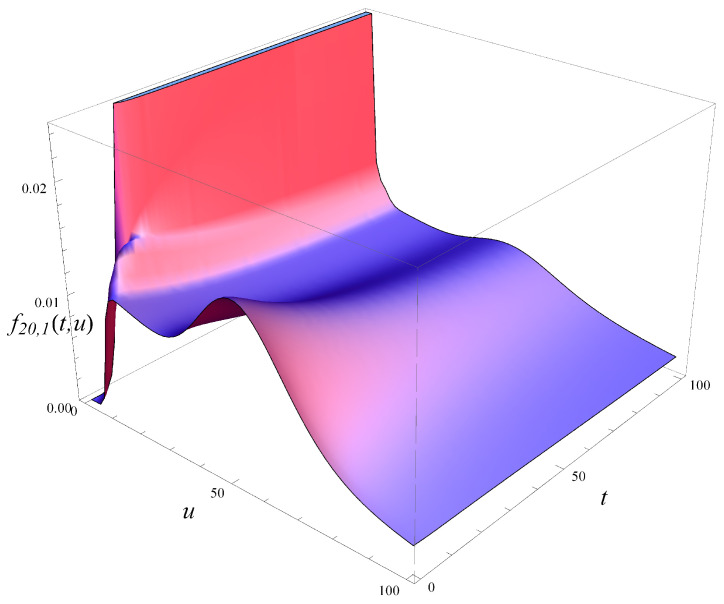
Probability density of workload in time. X(0)=20, S(0)=1.

**Figure 9:**
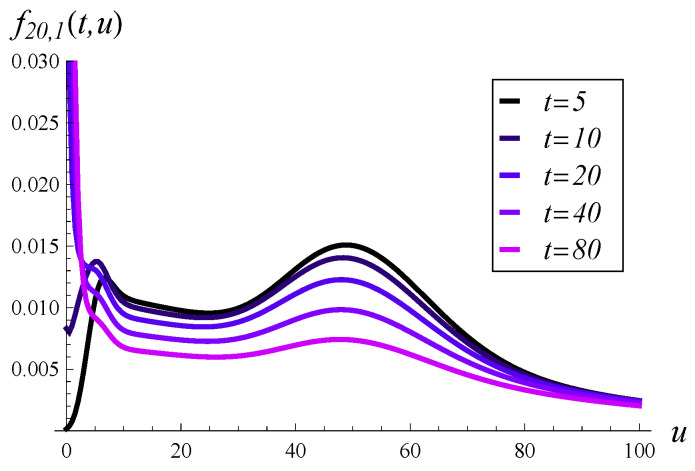
Probability density of workload in selected moments. X(0)=20, S(0)=1.

In Figure 8, the probability density of workload is depicted over time, now for an initially full system. Five vertical slices of Figure 8, taken at different moments in time, are shown in Figure 9. As seen in these figures, the density function can assume a complicated shape, with multiple extrema. For a small *t*, there is no probability mass near u=0, as the system occupancy is non-zero with a probability close to 1. However, this changes quickly with time.

**Figure 10:**
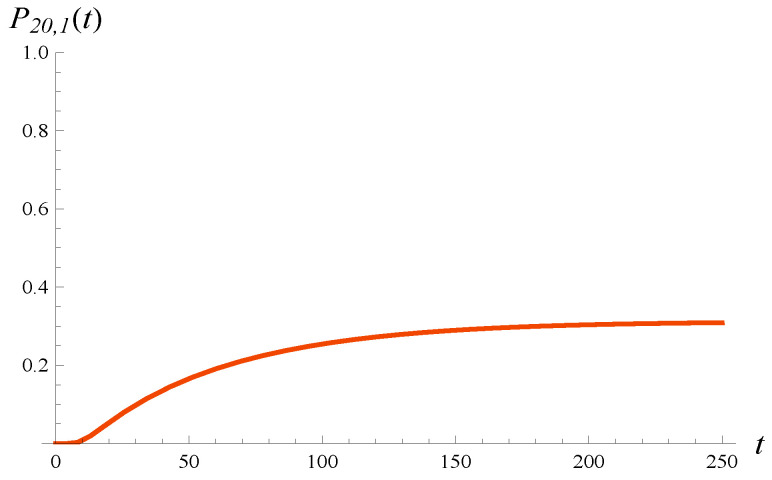
Probability that workload is zero versus time. X(0)=20, S(0)=1.

Finally, in Figure 10, the probability that the workload is zero, which is the missing probability mass in the defective density of workload, is depicted over time. Now the evolution of $P_{0,1}\left(t\right)$ is monotonic. It obviously converges to the same steady-state value as in Figure 7, i.e., 0.312.

## 7. Conclusions

We studied the queueing model with autocorrelated service times with respect to workload. The study was motivated by real queueing systems, in which such autocorrelation is quite common. The model we studied was universal in the meaning that the interarrival time distribution was general, while the service times were modeled by the Markovian service process, which allowed aligning the autocorrelation function of the model with an empirical autocorrelation function.

New formulas for the probability density of workload, its tail, entropy, and the average value were derived and illustrated through numeric examples, covering both time-dependent and steady-state workload of the system.

Among other findings, it was demonstrated that, in the model in question, workload can assume surprisingly large values, exceeding several times the product of the queue size and the average service time. This observation highlights the need to calculate workload as a separate characteristic. The queue size alone provides no insight into workload, as one might naively assume.

## Data Availability

Data is contained within the article.

## References

[1] Bocharov P.P. (1996). Stationary distribution of a finite queue with recurrent flow and Markovian service. Autom. Remote Control..

[2] Alfa A.S., Xue J., Ye Q. (2000). Perturbation theory for the asymptotic decay rates in the queues with Markovian arrival process and/or Markovian service process. Queueing Syst..

[3] Chaplygin V.V. (2003). The mass-service G/BMSP/1/r. Inf. Process..

[4] Bocharov P.P., D’Apice C., Pechinkin A.V., Salerno S. (2003). The stationary characteristics of the G/MSP/1/r queueing system. Autom. Remote Control..

[5] Albores-Velasco F.J., Tajonar-Sanabria F.S. (2004). Analysis of the GI/MSP/c/r queueing system. Inf. Process..

[6] Kim J., Kim B. (2007). Asymptotic analysis for loss probability of queues with finite GI/M/1 type structure. Queueing Syst..

[7] Gupta U.C., Banik A.D. (2007). Complete analysis of finite and infinite buffer GI/MSP/1 queue—A computational approach. Oper. Res. Lett..

[8] Banik A.D., Gupta U.C. (2007). Analyzing the finite buffer batch arrival queue under Markovian service process: *GI*^X^/*MSP*/1/*N*. Top.

[9] Banik A.D. (2011). Analyzing state-dependent arrival in GI/BMSP/1/*∞* queues. Math. Comput. Model..

[10] Chaudhry M.L., Samanta S.K., Pacheco A. (2012). Analytically explicit results for the GI/C-MSP/1/*∞* queueing system using roots. Probab. Eng. Informational Sci..

[11] Samanta S.K. (2015). Sojourn-time distribution of the GI/MSP/1 queueing system. Opsearch.

[12] Chaudhry M.L., Banik A.D., Pacheco A. (2017). A simple analysis of the batch arrival queue with infinite-buffer and Markovian service process using roots method: *GI*^X^/C-MSP/1/∞. Ann. Oper. Res..

[13] Banik A.D., Chaudhry M.L., Kim J.J. (2018). A Note on the Waiting-Time Distribution in an Infinite-Buffer *GI*^[^*X*]/C-MSP/1 Queueing System. J. Probab. Stat..

[14] Banik A.D., Ghosh S., Chaudhry M.L. (2019). On the consecutive customer loss probabilities in a finite-buffer renewal batch input queue with different batch acceptance/rejection strategies under non-renewal service. Soft Computing for Problem Solving: SocProS 2017.

[15] Banik A.D., Ghosh S., Chaudhry M.L. (2020). On the optimal control of loss probability and profit in a GI/C-BMSP/1/N queueing system. Opsearch.

[16] Samanta S.K., Bank B. (2021). Modelling and Analysis of GI/BMSP/1 Queueing System. Bull. Malays. Math. Sci. Soc..

[17] Chydzinski A. (2024). Transient GI/MSP/1/N Queue. Entropy.

[18] Ozawa T. (2004). Analysis of Queues with Markovian Service Processes. Stoch. Model..

[19] Zhang Q., Heindl A., Smirni E. (2005). Characterizing the BMAP/MAP/1 departure process via the ETAQA truncation. Stoch. Models.

[20] Horváth A., Horváth G., Telek M.A. (2010). Joint moments based analysis of networks of MAP/MAP/1 queues. Perform. Eval..

[21] Wang Y.C., Chou J.H., Wang S.Y. (2011). Loss pattern of D-BMAP/D-MSP/1/K queue and its application in wireless local communications. Appl. Math. Modell..

[22] Sandhya R., Sundar V., Rama G., Ramshankar R., Ramanarayanan R. (2015). BMAP/BMSP/1 queue with randomly varying environment. ISOR J. Eng..

[23] Samanta S.K., Chaudhry M.L., Pacheco A. (2016). Analysis of BMAP/MSP/1 queue. Methodol. Comput. Appl. Probab..

[24] Ghosh S., Banik A.D. (2017). An algorithmic analysis of the BMAP/MSP/1 generalized processor-sharing queue. Comput. Oper. Res..

[25] Ghosh S., Banik A.D. (2018). Computing conditional sojourn time of a randomly chosen tagged customer in a BMAP/MSP/1 queue under random order service discipline. Ann. Oper. Res..

[26] Nandi R., Samanta S.K. (2020). Stationary analysis of an infinite-buffer D-MAP/D-MSP/1 queueing system. Am. J. Math. Manag. Sci..

[27] Samanta S.K., Das K. (2023). Detailed analytical and computational studies of D-BMAP/D-BMSP/1 queueing system. Methodol. Comput. Appl. Probab..

[28] Chydzinski A., Adamczyk B. (2012). Transient and stationary losses in a finite-buffer queue with batch arrivals. Math. Probl. Eng..

[29] Mrozowski P., Chydzinski A. (2018). Queues with Dropping Functions and Autocorrelated Arrivals. Methodol. Comput. Appl. Probab..

[30] Latouche G., Ramaswami V. (1999). Introduction to Matrix Analytic Methods in Stochastic Modeling.

[31] Dudin A.N., Klimenok V.I., Vishnevsky V.M. (2020). The Theory of Queuing Systems with Correlated Flows.

[32] Chydzinski A. (2007). Time to reach buffer capacity in a BMAP queue. Stoch. Model..

[33] Politis D.N. (1994). Maximum Entropy Modelling of Mixture Distributions. Kybernetes.

[34] Cohen A.M. (2007). Numerical Methods for Laplace Transform Inversion.

